# La pseudarthrose du col fémoral traitée par prothèse totale de la hanche: à propos de 15 cas

**DOI:** 10.11604/pamj.2014.19.58.5102

**Published:** 2014-09-23

**Authors:** Aniss Chagou, Réda Allah Bassir, Abdelkarim Rhanim, Abdou Lahlou, Ahmed Bardouni, Moustapha Mahfoud, Mohammed Saleh Berrada, Moradh El Yaacoubi

**Affiliations:** 1 Service de Traumatologie-Orthopédie, Centre Hospitalier Universitaire Rabat, Rabat, Maroc

**Keywords:** Pseudarthrose du col fémoral, nécrose tête fémorale, arthroplastie de la hanche, Pseudoarthrosis of femoral neck, femoral head necrosis, hip replacement

## Abstract

La pseudarthrose du col fémoral est une complication redoutée de la facture du col fémoral, elle est due soit à une négligence thérapeutique, soit à une ostéosynthèse imparfaite. Plusieurs facteurs sont incriminés dans sa genèse, l’âge, les caractéristiques de la fracture, l’état de la tête fémorale, et une ostéosynthèse non solide. Le diagnostic des pseudarthroses est essentiellement radiologique. Le traitement reste encore difficile et mal codifié, les limites entre traitement conservateur et arthroplastie de la hanche ne sont pas encore bien définies. Nous rapportons une série de 15 cas de pseudarthrose du col fémoral traités par arthroplastie de la hanche au service de traumato-orthopédie du Centre Hospitalier Universitaire de Rabat de 2008 à 2014 soit un recul de 40 mois en moyenne. Nos patients ont bénéficié d'une évaluation clinique et radiologique. L’âge de nos patients varie entre 48 et 81 ans, avec une moyenne de 69,2 ans. 85% d'entre eux sont âgés de plus de 60 ans. Nous avons dans notre série une prédominance féminine, soit 8 femmes pour 7 hommes. La négligence thérapeutique est la cause de la majorité des pseudarthroses du col du fémur traitées dans notre série. Par ailleurs, nous avons utilisé exclusivement la voie d'abord postéro externe de Moore. Nous avons mis en place une prothèse totale de couple polyéthylène-métal chez tous les patients, ces prothèses étaient cimentées chez 12 patients. Le résultat fonctionnel a été coté selon la classification de Merle d'Aubigné. Nos résultats ont été jugés bons selon cette cotation. L'arthroplastie totale de la hanche a une place importante dans le traitement des pseudarthroses du col fémoral et peut donner des résultats satisfaisants en permettant de récupérer une hanche mobile et indolore.

## Introduction

La pseudarthrose du col fémoral est une complication assez fréquente de la fracture du col fémoral. Elle est définie par l'absence ou l'arrêt de consolidation d'une fracture cervicale vraie. Différentes techniques chirurgicales sont indiquées en l'occurrence l'ostéotomie de valgisation [1–4], la greffe pédiculée de Judet [5] et l'arthroplastie totale de la hanche [6–9]. L'indication thérapeutique dépend de l’âge physiologique, du type de pseudarthrose, et de la vitalité de la tête fémorale. Si les deux premiers points sont faciles à résoudre, le troisième est beaucoup plus ardu et explique que les indications thérapeutiques sont pleines d'aléas. A travers 15 cas de pseudarthroses du col du fémur traités par arthroplastie de la hanche opérés au centre hospitalier universitaire de Rabat de 2008 à 2014, nous discuterons les arguments cliniques et radiologiques qui nous ont poussés à poser l'indication de l'arthroplastie totale de la hanche. Nous évaluerons également son taux de réussite par rapport au traitement conservateur.

## Méthodes

Nous rapportons une étude rétrospective centrée sur 15 cas de pseudarthrose du col fémoral traités par arthroplastie totale au centre hospitalier de Rabat entre 2008 et 2014 avec un recul moyen de 38 mois. Le suivi des patients a été clinique et radiologique.

## Résultats

**Figure 1:**
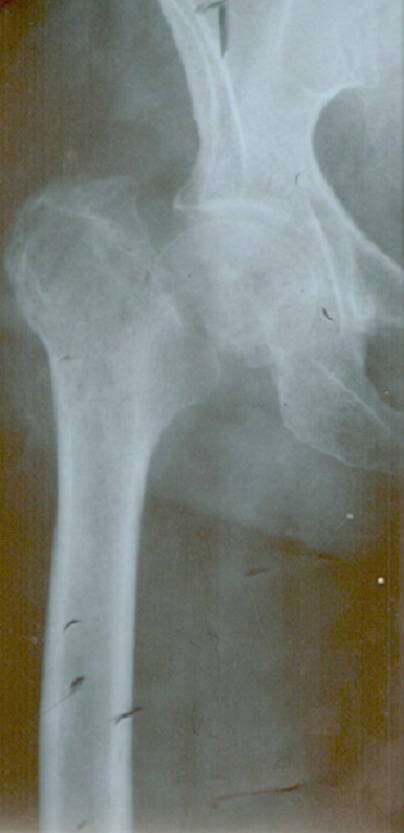
Radiographie de hanche de face montrant une pseudarthrose du col suite à une fracture negligee

**Figure 2:**
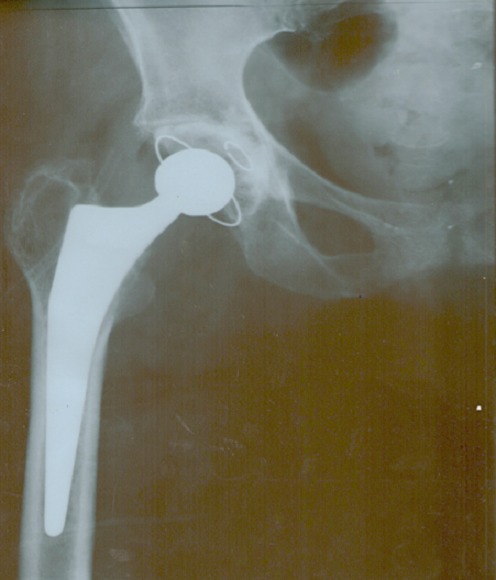
Radiographie montrant une pseudarthrose après traitement par vissage chez un homme de 54 ans

L’âge de nos patients varie entre 48 et 81 ans, avec une moyenne de 69,2 ans. 85% d'entre eux sont âgés de plus de 60 ans. Nous avons dans notre série une prédominance féminine, soit 8 femmes pour 6 hommes. Une chute domestique de leur hauteur a été la cause de l'accident chez 11 patients, alors que 4 patients sont victimes d'accidents de la voie publique et sont les patients les plus jeunes de la série. Dans notre série, le délai entre la fracture initiale et la consultation pour une prise en charge varie entre 3 et 18 mois. La négligence thérapeutique est la cause de la majorité des pseudarthroses du col du fémur traitées dans notre série (Figure 1). En effet, 12 patients parmi nos 15 cas, n'ont reçu aucun traitement, leur âge était compris entre 48 entre et 81 ans. Pour la majorité (9 patients), ces patients n'ont consulté que tardivement pour des douleurs de la hanche ou une boiterie alors que deux patients ont essayé un traitement dans une structure non médicale. Deux patients ont bénéficié d'un traitement chirurgical initial, il s'agit d'ostéosynthèse par vissage (Figure 2). Le délai entre ce dernier et la mise en place de la prothèse est de 6 et 18 mois. Cliniquement, la majorité des malades présentait une impotence fonctionnelle partielle ou totale. Cette dernière les avait obligé à rester alité pour la moitié des patients, alors que la marche était possible pour les autres à l'aide d'une ou de deux cannes. La boiterie était présente chez 13 patients. L'examen de nos malades nous a permis de retrouver un certain nombre de caractéristiques propre à la pseudarthrose, un raccourcissement net du membre atteint qui variait de 2 à 4 cm, une amyotrophie qui a été notée dans tous les cas de notre série. La mobilité de la hanche atteinte est limitée chez la quasi-totalité de nos patients, tous les mouvements sont plus au moins affectés. Sur le plan radiologique, Nous avons relevé 11 cas de pseudarthrose lâche contre 4 cas de pseudarthrose serrée. La pseudarthrose siégeait au niveau trans-cervical dans 8 cas et basicervical dans 7 cas. Dans tous le cas, nous avons constaté sur les radiographies du bassin de face, une ascension nette du grand et du petit trochanter au niveau du côté atteint. Dix patients présentaient une déviation de l'axe fémoral dont neuf en adduction et un en abduction.

Pour la totalité de nos patients la tête fémorale était en place. La radiographie standard ne peut juger de son état vasculaire, et aucun de nos malades n'a bénéficié d'une scintigraphie ou d'IRM. On a noté chez deux de nos patients la présence sur la radiographie du bassin de face, la présence de quelque débris osseux autour de la tête fémorale. La minéralisation osseuse a été jugée sur des radiographies standards, ce critère est d'une valeur importante car il témoigne de la fragilité osseuse qui favorise la survenue de la fracture du col. Dix patients présentaient une ostéoporose qui était nette sur la radiographie standard, les patients restants avaient une minéralisation osseuse normale. Aucun de nos malades n'a réalisé l'ostéodensitomètrie.

**Figure 3:**
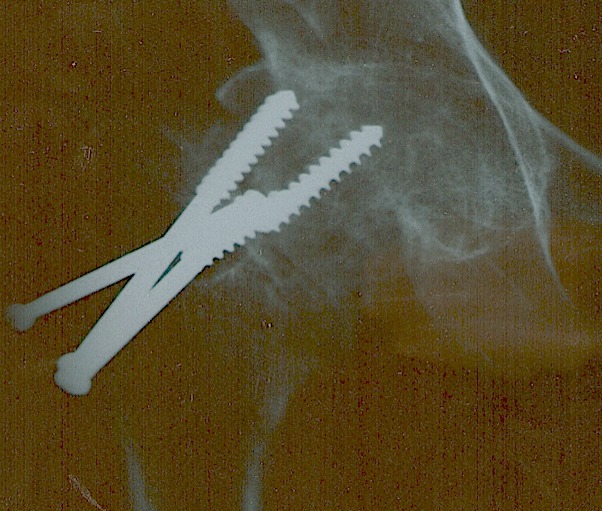
Radiographie de la hanche de face après arthroplastie totale de la hanche

**Figure 4:**
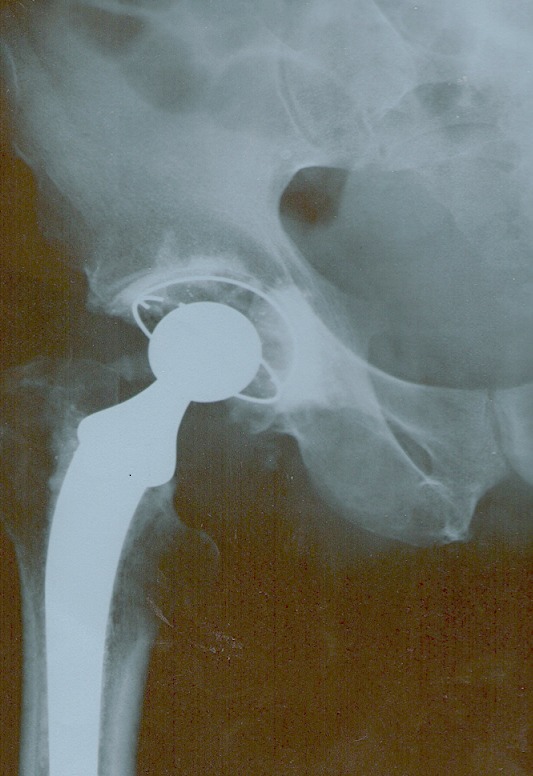
Radiographie post-opératoire de la hanche du 2ème patient

Par ailleurs, nous avons utilisé exclusivement la voie d'abord postéro externe de Moore. Nous avons mis en place une prothèse totale de couple polyéthylène-métal chez tous les patients (Figure 3, Figure 4), ces prothèses étaient cimentées chez 13 patients. Les deux patients les plus jeunes de la série 48 et 54 ans ont bénéficié de prothèses non cimentées. Les patients ont été mis sous anti-coagulation et antibiothérapie préventives. L'appui et la rééducation ont été commencés le lendemain. Le recul moyen est de 36 mois (de 6 mois à 48 mois), Les résultats fonctionnels ont été évalués selon la cotation de Merle d'Aubigné [9] qui se base sur l'appréciation de la douleur, la mobilité et la marche (Tableau 1). Huit patients n'ont jamais signalé de douleur ni à la marche ni à la reprise de l'appui, ni lors de la révision. La récupération des amplitudes de l'articulation coxo-fémorale était très satisfaisantes chez douze des quatorze malades, avec une amplitude de flexion qui allait de 90° à 120°. Deux malades avaient une mobilité réduite. L'appui monopodal était stable dans la majorité des cas avec stabilité parfaite et marche normale. La récupération de la force musculaire a été progressive avec les séances de rééducation et une réadaptation à la marche. Nos résultats sont synthétisés dans le (Tableau 2). Ainsi, la moyenne des résultats sur la douleur, la mobilité, et la marche donne une cotation à 16,07. Les résultats de notre série selon cette cotation sont jugés bons.

**Tableau 1:** Cotation de Merle d'Aubigné, qui met l'accent sur 3 points essentiels: la douleur, la mobilité, la marche

Cotation	Douleur	Mobilité	Marche
0	Douleur très vive et continue	Ankylose en attitude vicieuse	Impossible
1	Douleur très vive empêchant le sommeil	Ankylose clinique sans attitude vicieuse	Avec 2 béquilles
2	Douleur très vive à la marche empêchant toute activité	Flexion 40° Abduction 0° Attitude vicieuse légère	Avec 2cannes
3	Douleur vive après ¼ H de marche	Flexion de 40à60°	Limitée avec canne, impossible sans canne
4	Douleur après 4 H de marche, disparaît au repos	Flexion 60 à 80°, peut placer sa chaussure	Prolongée avec canne, limitée sans canne
5	Douleur au démarrage	Flexion 80 à 90° abduction 25°	Sans canne, claudication légère
6	Indolence complète	Flexion 90° abduction 40°	Normale

**Tableau 2:** Nos résultats selon la cotation de Merle d'Aubigné

	Douleur	Mobilité	Marche
1	-	-	-
2	-	-	-
3	1 cas	2 cas	-
4	-	2 cas	3 cas
5	6 cas	4 cas	2 cas
6	8 cas	7 cas	10 cas

La surveillance radiologique a été simple, limitée à un cliché du bassin face, une hanche et profil prenant la totalité de la prothèse. L’étude soigneuse des radiographies successives et leur confrontation avec le cliché post opératoire qui nous a servi de référence tout au long de l’évolution de la prothèse. Ainsi la jonction os-ciment a été analysée zone par zone, les liserés ont été recherchés dans chaque zone de l'interface os-ciment. L'environnement de la prothèse a été étudié selon le schéma établi par Frot; La position de pièces cotyloïdiennes a été surveillée selon la méthode de Cheurot. Nous n'avons pas noté de cas de descellements francs ou de liserés pouvant faire suspecter un descellement.

## Discussion

Nous avons remarqué dans notre série, la fréquence augmentée des pseudarthroses sur fractures du col fémoral non traitées ou traitées par des méthodes non médicales (12 cas). Dans notre pays la fréquence de la pseudarthrose du col fémorale reste encore élevée et mal évaluée, du fait du recours à ce genre de traitement. La fréquence de cette complication est variable entre 4% et 53% selon les auteurs. L’âge moyen des patients de notre série au moment de l'arthroplastie de la hanche est de 69,2 ans. Dans la série Zehi [6], l’âge moyen est de 44.5 ans. Dans notre étude il y avait une légère prédominance féminine: 8 femmes et 7 hommes, ce qui concorde avec la série de Pidhorz [7], qui comptait 32 femmes et 22 hommes. En ce qui concerne le siège de la pseudarthrose on a souligné la fréquence de le forme trans-cervicale 57%, ces résultats sont comparables a ceux d'autres auteurs (Tableau 3). Tous les foyers de pseudarthrose étaient accompagnés d'une ascension du grand trochanter, et une résorption du col fémoral.

**Tableau 3:** Siège de la pseudarthrose dans les différentes séries, comparaison de nos résultats avec celle de Zehi [6], Pidhorz [7], Decoulx [10]

	Nombre	Trans-cervicale	Basi-cervicale	Sous capitale
PIdhorz [7]	53 cas	47%	13%	40%
Zehi [6]	41 cas	40%	50%	10%
Decoulx [10]	42 cas	57%	32%	11%
Notre série	14 cas	57%	36%	7%

Par ailleurs, la tête fémorale était en place mais on n'a pas pu juger de sa viabilité chez tous les patients, on n'a réalisé ni scintigraphie ni IRM. Chez deux patients la nécrose était bien évidente sur la radiographie du bassin face. Zehi [6] a trouvé 9 cas de nécrose de la tête, mais sur une série de 41 cas. La PTH a été utilisée dans notre étude après échec de traitement initial (vissage) dans 2 cas et sur une fracture négligée dans 13 cas. Dans la série de Pidhorz [7] l'arthroplastie a été indiquée après échec du traitement conservateur dans 9 cas, et sur une fracture négligée dans 12 cas. La durée entre le traitement initial, et l'apparition de la non consolidation a varié dans notre série entre 6 mois et 18 mois. Dans la même série de Pidhorz cette durée a variée entre 8 mois et 5 ans.

Peu d'articles discutent les résultats d'arthroplasties de la hanche dont l'indication est posée après échec du traitement conservateur ou suite à une fracture négligée [6, 7, 8, 10]. Même si nos résultats sont globalement satisfaisants, le recul insuffisant rend difficile une estimation crédible que ce soit sur le plan fonctionnel où nous avons un score de 16,07 sur la cotation de Merle d'Aubigné ou radiologiquement où nous n'avons relevé aucun descellement radiologique, Marbry [8] a rapporté 9 cas de descellement septique et aseptique dans sa série faite de 99 cas mais avec un recul de 10 ans.

## Conclusion

La pseudarthrose du col fémoral est une complication assez fréquente et toujours redoutée devant la facture du col fémoral; son traitement n'est pas encore bien codifié. L'arthroplastie totale de la hanche a une place importante et peut donner des résultats satisfaisants en permettant de récupérer une hanche mobile et indolore.
